# Genetic diversity assessment of *Vitis ficifolia* Bge. populations from Henan province of China by SRAP markers

**DOI:** 10.1080/13102818.2014.984414

**Published:** 2014-12-03

**Authors:** Xiucai Fan, Jianfu Jiang, Ying Zhang, Haisheng Sun, Jian Jiao, Chonghuai Liu

**Affiliations:** ^a^ Zhengzhou Fruit Research Institute, Chinese Academy of Agricultural Sciences, Zhengzhou, Henan, P.R. China

**Keywords:** genetic diversity, *Vitis ficifolia* Bge., populations, SRAP

## Abstract

Eighteen sequence-related amplified polymorphism (SRAP) primer combinations were used to assess the genetic diversity of 126 individuals from five different geographical populations of *Vitis ficifolia* Bge. The numbers of bands scored per primer combination ranged from 8 to 27, with an average of 18.6 bands. At the population level, the percentage of polymorphic bands (PPB), Nei's gene diversity index (*H*) and Shannon's information index (*I*) were the highest in the Shihe (Xinyang) population (77.31%, 0.1987, 0.2805) and the lowest in the Linzhou (Anyang) population (55.82%, 0.1112, 0.1727). At the species level, PPB, *H* and *I* were 80.56%, 0.2129 and 0.3075, respectively. The genetic differentiation coefficient (*G*_ST_) was 0.2055 and the gene flow (*N_m_*) was 1.9328, indicating strong intra-population genetic differentiation. Based on the unweighted pair group method based arithmetic average clustering diagram, the five studied populations may be divided into three groups. The clustering results were almost in accordance with the populations’ geographical distribution.

## Introduction

The genetic diversity of plant species is a major concern for geneticists and plant breeders.[1] The current methods for measuring genetic diversity of populations and germplasm collections are often conducted through statistics calculated from molecular marker data.[2] Analysis of the genetic structure at an intra-specific level is important for better understanding of future adaptive changes or evolution,[3] and also for future breeding programmes.

Grapevine (*Vitis* spp.) is one of the most important fruit crops worldwide with more than 70 species of *Vitis* spp. in the world.[4–6] More than 38 *Vitis* species have their origin in China, making China one of the major gene centres of *Vitis* species origination.[5] Chinese wild grape species are of particular interest in grape breeding for their desirable characteristics, such as disease resistance, drought tolerance and cold hardiness genes.[5,7,8] *V. ficifolia* Bge. is a subspecies of *V. heyneana* Roem. et Schult, and is distributed mainly in the northern and eastern China, at altitudes between 100 and 1300 m,[5] especially in Henan Province in Central China. *V. ficifolia* has excellent tolerance to anthracnose, ripe rot diseases and winter cold, and is used both as table grape and in wine production.

One of the most powerful tools for analysis of genomes is molecular genetic markers. They allow for heritable traits to be associated with underlying genomic variation.[9] Sequence-related amplified polymorphism (SRAP) is a polymerase chain reaction (PCR)-based marker system targeting open reading frames (ORFs).[10] With reproducible results, high reliability, simple technology and low cost, SRAP has been applied in the assessment of the population structure and genetic diversity of many fruit species.[11–18]

In this study, SRAP analysis was employed to investigate: (1) the levels of SRAP variations in *V. ficifolia* Bge. populations; (2) the levels of genetic diversity within and among populations and (3) the genetic relationships among populations.

## Materials and methods

### Plant materials

**Figure 1.**
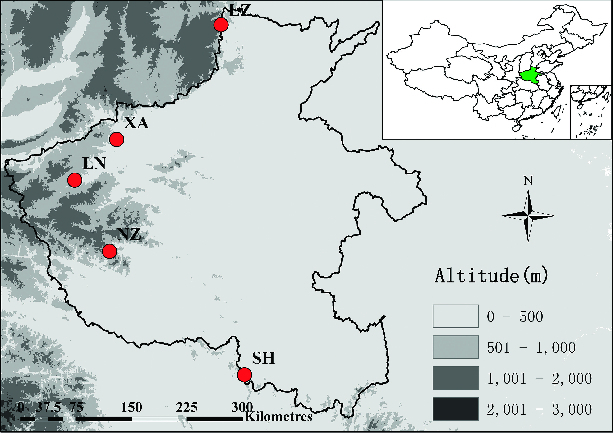
Map showing locations of the sampled populations of *V. ficifolia* Bge.

A total of 126 *V. ficifolia* Bge. individuals of five different wild populations were selected from Henan Province of China. Their places of origin and distributions are shown in Table 1 and Figure 1.

**Table 1.** Different locations of sample collection in Henan Province of China.

Population code	Locality	Sample size	Longitude E	Latitude N
SH	Shihe, Xinyang	29	113° 90′	31° 96′
NZ	Nanzhao County, Nanyang	25	111° 98′	33° 49′
LN	Luoning, Luoyang	24	111° 48′	34° 35′
XA	Xinan, Luoyang	26	112° 12′	34° 83′
LZ	Linzhou, Anyang	22	113° 75′	36° 07′

### DNA isolation

Total genomic DNA was isolated from fresh leaves following the procedure previously described by Liu et al. [16]. The quality and concentration of the DNA samples were checked in a biophotometer plus (Eppendorf, Germany) and a portion of the DNA was diluted to 50 ng/μL for subsequent SRAP analysis. Both the stock and diluted portions were stored at −20 °C.

### SRAP analysis

The SRAP analysis was carried out using 26 primers including 13 forward (Me1-13) and 13 reverse (Em1-13) SRAP primers 11-18,[10] for a total of 169 primer combinations. The primer sequences were synthesized by Shanghai Biological Engineering Technology and Service Co. Ltd. Each 10 μL PCR reaction mixture consisted of 30–50 ng of template DNA, 0.2 mmol/L of deoxynucleoside triphosphates (dNTPs), 1.0 μmol/L of each primer, 2.0 mmol/L of Mg^2+^ and 1 U Taq DNA polymerase (TaKaRa). The amplification reaction procedure was as follows: after denaturation at 94 °C for 5 min, the reaction mixture was subjected to amplification for seven cycles consisting of 90 s at 94 °C, 75 s at 35 °C, 30 s at 72 °C, followed by 30 cycles consisting of 60 s at 94 °C, 60 s at 55 °C and 90 s at 72 °C, with a final extension at 72 °C for 5 min.

### Data analysis

Amplified bands were scored as either present (1) or absent (0). The data were entered into a binary matrix and, subsequently, POPGENE 32 software was used to compute the population genetic parameters, the percentage of polymorphic bands (PPB), Shannon's information index (*I*), Nei's gene diversity (*H*), genetic identity and genetic distance, etc. AMOVA 30 software was used to estimate the relative genetic differentiation coefficient (*G*_ST_) and the level of gene flow (*N_m_*). Cluster analyses were conducted on the similarity matrix with the unweighted pair group method based arithmetic average (UPGMA), and the resulting clusters were expressed as dendrograms.

## Results and discussion

### SRAP polymorphism

Eighteen informative primers were selected due to their ability to produce clearly and repeatedly polymorphic and unambiguous bands among the accessions (Table 2). As shown in Table 3, a total of 335 bands were obtained with 18 primer combinations. Of these bands, 268 were highly polymorphic (80.56% polymorphism), ranging from 50 to 1500 bp. The numbers of amplicons scored per primer combination ranged from 8 (for combination Me10/Em13) to 27 (for combinations Me10/Em6 and Me10/Em8), with an average of 18.6 bands. The number of polymorphic bands (NPB) per primer combination ranged from 6 to 22; the average one was 14.9. The PPB for each primer combination varied from 59.26% (Me10/Em8) to 88.24% (Me4/Em6). The PPB of the Me4/Em6 primer combination in XA, NZ and SH populations was the highest (100.0%), and in LZ, the lowest (52.9%). In the five populations, SH was the highest with 17.3 average polymorphic bands and 77.31% PPB.

**Table 2.** Polymorphism revealed by different primer combinations used in SRAP analysis.

Primer combinations	TNB^a^	NPB^b^	PPB^c^ (%)	Size (bp)
Me1/Em6	18	14	77.77	50–1500
Me1/Em8	21	18	85.71	50–1500
Me2/Em8	22	19	86.36	50–1500
Me3/Em7	18	15	83.33	50–1200
Me3/Em8	20	17	85.00	50–1100
Me4/Em6	17	15	88.24	50–1500
Me5/Em6	23	19	82.61	100–1500
Me9/Em6	22	15	68.18	50–1000
Me9/Em13	16	13	81.25	50–800
Me10/Em6	27	22	81.48	100–1000
Me10/Em8	27	16	59.26	150–1500
Me10/Em13	8	6	75.00	150–900
Me11/Em6	13	11	84.62	110–1000
Me11/Em7	13	11	84.62	70–1500
Me11/Em8	20	17	85.00	50–1200
Me12/Em1	15	12	80.00	50–1200
Me13/Em7	20	15	75.00	100–1400
Me13/Em13	15	13	86.67	50–700
				
Mean	18.6	14.9	80.56	
Total	335	268		

Notes:

^a^TNB = number of total bands.

^b^NPB = number of polymorphic bands.

^c^PPB = percentage of polymorphic bands.

**Table 3.** Genetic diversity of different populations.

Population code	NPB^a^	PPB^b^ (%)	*H*^c^	*I*^d^
LZ	187	55.82	0.1112	0.1727
XA	227	67.76	0.1617	0.2335
LN	211	62.99	0.1792	0.2453
NZ	258	77.01	0.1963	0.2689
SH	259	77.31	0.1987	0.2805
				
Mean	228	68.18	0.1694	0.2402
Species level	268	80.56	0.2129	0.3075

Notes;

^a^NPB = number of polymorphic bands.

^b^PPB = percentage of polymorphic bands.

^c^ *H* = Nei's gene diversity.

^d^ *I* = Shannon's information index.

SRAP was initially developed for *Brassica* species, and was tested in other crops. In genetic diversity analysis, the information given by SRAP markers is concordant with the morphological variability.[16,19] The detection of high levels of polymorphism makes SRAP analysis a powerful tool for assessment of genetic diversity in many species.[15,20,21] To the best of our knowledge, the present study is the first report of genetic investigation of *V. ficifolia* Bge. using SRAP markers. The results indicated that SRAP markers could be used efficiently in the genetic diversity and genetic variability of *V. ficifolia* Bge.

### Genetic diversity

Genetic polymorphism may be indicative of evolutionary adaptation which plays a key role for survival of a population in the changing environment.[22] Accurate estimates of genetic diversity are particularly useful for optimization of sampling strategies and for conservation and management of the genetic diversity of trees.[23] The estimates of the genetic diversity in each population are summarized in Table 3. The percentage of polymorphic bands ranged from 55.82% (LZ) to 77.31% (SH). Nei's gene diversity index of the five populations ranged from 0.1112 to 0.1987 and Shannon's diversity index ranged from 0.1727 to 0.2805. Among these five populations, populations SH and NZ exhibited the highest level of variability (PPB: 77.31% and 77.01%, *H*: 0.1987 and 0.1963, *I*: 0.2805 and 0.2689, respectively), whereas population LZ exhibited the lowest level of variability (PPB: 55.82%, *H*: 0.1112, *I*: 0.1727), as shown in Table 3.

### Genetic differentiation and gene flow

Genetic differentiation and gene flow are important indices for evaluation of the genetic structure of a group. Based on the analysis by AMOVA 30 software, the relative genetic differentiation coefficient (*G*_ST_) was 0.2055, which showed that the major genetic variation (72.95%) originated from intra-population diversity, and the rest (20.55%) existed among populations. The level of gene flow (*N_m_*) is a key factor that affects the genetic structure and genetic differentiation among populations. Based on the AMOVA analysis, the level of gene flow (*N_m_*) among the five populations was 1.9328.

The results obtained here showed that a high proportion of the variability was due to intra-population variability, and this high genetic variability was consistent with the previous results obtained by morphological analysis.[24] Gene flow is reversely correlated with the group genetic differentiation. Also, gene flow is very important for the dispersal and evolution of plant populations. In seed plants, gene flow is mainly achieved by seeds or pollen carrying foreign genes between groups.[25] When *N_m_* > 1.0, this indicates that the level of gene flow is higher; when *N_m_* > 5.0, the outcrossing rate is higher.[26] In this study, the gene flow among populations was high (*N_m_* = 1.9328). This indicated that high-level gene flow existed within *V. ficifolia* populations. This result was consistent with the previous results.[22,27–31] The reason for slightly higher *G*_ST_ may lie in the morphological character of the wild *V. ficifolia*, with the vines growing up into tall plants, and the tall plants benefitting due to reduced resistance to pollen movement in the air. Moreover, a lack of effective geographic isolation may also contribute to the improvement of gene homogenization among populations.[32] Widely distributed plant species must adapt to a broad range of environmental conditions to maintain their large geographic distributions.[33] Consequently, many widespread species have high genetic diversity or exhibit considerable phenotypic plasticity.[34,35] *V. ficifolia* has wide distribution in northern and eastern China, especially in Henan Province. However, it should be noted that the *V. ficifolia* populations used in this study were sampled from a narrow eco-geographical area of distribution.

### Genetic similarity

In genetic diversity analysis, there are various genetic distance measures for analysis of molecular marker data. Genetic identity is an important index for estimation of the genetic differentiation among populations. Based on our POPGENE analysis, the genetic identity and genetic distance between each two populations, respectively, ranged from 0.9171 to 0.9825 and from 0.0176 to 0.0866. This suggested that there was a high genetic similarity between the populations, indicating lower reproductive isolation among the populations (Table 4). The minimum genetic identity occurred between SH and LZ; the maximum one occurred between LN and XA; and SH population constituted the farthest genetic relationship with the other four populations.

**Table 4.** Genetic identity and genetic distance of the five studied populations.

Population code	SH	NZ	LN	XA	LZ
SH	–	0.9641	0.9556	0.9353	0.9171
NZ	0.0368	–	0.9729	0.9699	0.9512
LN	0.0455	0.0275	–	0.9825	0.9650
XA	0.0669	0.0305	0.0176	–	0.9676
LZ	0.0866	0.0501	0.0356	0.0329	–

Note: Nei′s genetic identity (above diagonal) and genetic distance (below diagonal).

### Cluster analysis

**Figure 2.**
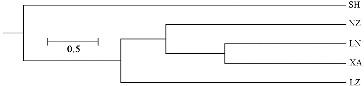
Dendrogram of the five geographical populations using UPGMA.

The dendrogram obtained with SRAP data is shown in Figure 2. The five populations may be divided into three groups. The populations located nearest to each other, NZ, XA and LN, were classified as the first group. Then, they clustered with population LZ, and population SH in southern Henan formed a separate group. The genetic relationships between populations of a widespread species often do not correspond to their geographical distance.[36,37] However, Ma et al. [33] found that most of the *Elymus sibiricus* populations studied by them clustered in accordance with the geographic distribution. In our study, the clustering results were almost in accordance with the geographical distribution.

The *in situ* method allows continuing evolution of the species in its natural habitat and then guaranteeing the maintenance of most of the species’ genetic variation. In this study, population SH showed relatively high genetic diversity within *V. ficifolia* Bge., and should therefore be a priority site for *in situ* conservation.

## Conclusions

The present study is, to the best of our knowledge, the first report of genetic investigation of *V. ficifolia* Bge., using SRAP markers. The results indicated that SRAP markers could be used efficiently in the study of genetic diversity and genetic variability of *V. ficifolia*. High-level gene flow existed within *V. ficifolia* populations. The clustering results based on SRAP were almost in accordance with the geographical distribution of the populations. In our study, the Shihe population (SH) showed relatively high genetic diversity within *V. ficifolia* Bge., and should therefore be a priority site for *in situ* conservation.
